# Analyzing the switch from laparoscopic to robotic surgery for diverticular disease: a comparative cohort study

**DOI:** 10.1007/s11701-025-03061-2

**Published:** 2025-12-22

**Authors:** Rahul Bhome, Ramprasad Rajebhosale, Rachel Smyth, Estelle Martin, Matthew Tutton, Subash P. Vasudevan

**Affiliations:** 1 Colorectal Unit, Colchester Hospital, East Suffolk and North Essex Foundation Trust, Turner Road, Colchester CO4 5JL, UK; 2 University Surgery, University of Southampton, Level C South Academic Block, Southampton General Hospital, Tremona Road, Southampton SO16 6YD, UK; 3 Colorectal Unit, University Hospitals Southampton NHS Trust, Tremona Road, Southampton SO16 6YD, UK; 4 Anglia Ruskin University, East Rd, Cambridge CB1 1PT, UK

**Keywords:** Robotic surgery, DaVinci, Diverticular disease, Elective, Left-sided, Anterior resection, Hartmann’s procedure

## Abstract

The aim of this study was to compare short term clinical outcomes for patients having robotic or laparoscopic diverticular resection in the elective setting. This was a single center cohort study of consecutive patients having elective minimally invasive left-sided diverticular resection between February 2018 and March 2025 for symptomatic inflammation, stricture, abscess or fistula. All robotic procedures were carried out using the DaVinci Xi system (Intuitive Surgical). The primary outcome was rate of open conversion. Secondary outcomes included operative time, blood loss, stoma formation, duration of patient-controlled analgesia, time to gut function, length of stay and complications. Seventy-two patients were included, 40 in the robotic group and 32 in the laparoscopic group. Open conversion rate, blood loss and patient-controlled analgesia use were significantly reduced in patients having robotic compared to laparoscopic surgery, at the cost of operative time. Robotic surgery was an independent predictor of lower conversion rate, lower blood loss and higher operative time in regression models. Robotic surgery for elective diverticular resection may enable a greater proportion of patients to benefit from minimally invasive approach without the need for open conversion.

## Introduction

Diverticular disease is the second most common indication for elective colonic resection [1]. Often, diverticular resections are challenging due to inflammation and fibrosis, which distort familiar tissue planes, increasing tissue trauma, blood loss and risk of injury to adjacent structures [2, 3]. For these reasons, the rates of open surgery for diverticular disease remained high in the laparoscopic era and conversion rates were up to 20% [4, 5]. Several meta-analyses suggest that robotic surgery significantly reduces the open conversion rate for diverticular resection compared to laparoscopic surgery [6–8]. This is important as it may allow a greater proportion of patients to benefit from faster recovery, reduced length of stay and reduced complication rates that the minimally invasive approach offers [5].

Recent guidelines recommend elective resection in patients with fistulation, stenosis, persistent abscess or in symptomatic patients with evidence of inflammation on imaging, as opposed to patients with recurrent episodes of acute uncomplicated diverticulitis [9–11]. Historically, elective surgery was offered more readily, after a second attack of acute diverticulitis for example, and as a spillover of this, the indication for elective surgery still varies significantly from unit to unit [12, 13]. For this reason, meta-analyses typically include patients with a broad and often poorly characterized disease phenotype. In addition to disease phenotype, we should be cautious about meta-analyzing studies which are heterogenous in terms of surgeon experience, surgical technique (including use of different robotic platforms), perioperative care and enhanced recovery protocols. In this regard, single center studies may have certain advantages in terms of greater uniformity of these parameters. Although single center studies have been published on this topic, to the best of our knowledge there are none from the UK. It is important to assess whether the benefits of robotic surgery hold true in different populations and in different healthcare systems.

Furthermore, it is important to highlight that observational studies remain relevant in surgery, particularly when comparing different surgical procedures, due to the challenges faced with randomized controlled trials (RCTs). These include the procedural learning curve, variation in technique, variation in quality of surgery, patients declining randomization and inherent problems with blinding [14]. Undoubtedly, randomization eliminates selection bias and balances confounding factors between groups, thereby reducing systematic error in comparison to observational studies, but RCTs are more difficult to conduct with surgical interventions. The IDEAL framework accepts that there is a place for observational studies alongside RCTs in the evaluation of new surgical procedures [15].

The aim of the present study was to assess the transition from laparoscopic to robotic surgery for diverticular disease in a UK center, where indication for surgery met current guidelines, disease phenotypes were well-defined, and surgical technique and peri-operative care were standardized.

## Methods

This was a retrospective analysis of a prospectively collected dataset. Consecutive patients who had elective minimally invasive (robotic or laparoscopic) left-sided diverticular resections between 1 st February 2018 and 30 th March 2025 at a single UK center (Colchester Hospital, East Suffolk and North Essex Foundation NHS Trust) were included. Indications for elective diverticular resection were: (i) ongoing symptoms with cross sectional or luminal imaging showing inflammation; (ii) stricture; (iii) abscess; or (iv) fistula. This was in accordance with latest ACPGBI and ESCP guidelines [9, 11]. Patients having planned open surgery were excluded as were those having emergency surgery. No patients were excluded based on age, BMI or American Society of Anesthesia (ASA) grade.

Data were procured from the Evolve medical record platform (Kainos, Belfast, UK). A database of prospectively collected anonymized data was securely stored in accordance with local governance protocols for statistical analysis. Demographic parameters included: age, gender, body mass index, smoking status, comorbidities (hypertension, ischemic heart disease, COPD, diabetes mellitus), ASA-grade, previous abdominal surgery and diverticular disease phenotype (symptomatic inflammation, stricture, abscess or fistula). Operative parameters included: conversion to open surgery, operative time (and docking time where applicable), blood loss (categorized < 100 ml, 100–500 ml, > 500 ml), stoma formation (end colostomy, defunctioning ileostomy). Post-operative parameters were duration of PCA, LOS, complications (Clavien-Dindo (CD) grade), RTT and 30-day re-admission. The primary outcome was conversion to open surgery. Secondary outcomes were operative time, blood loss, stoma formation, length of stay (LOS), admission to critical care, time to gut function, duration of patient-controlled analgesia (PCA), anastomotic leak, return to theatre (RTT), post-operative complications and 30-day re-admission.

Left sided diverticular resections included high anterior resection (with or without defunctioning ileostomy) and Hartmann’s procedure. All anastomoses (where appropriate) were fashioned using a circular trans-anal stapler (side to end or end to end). All patients having MIS were enrolled on an enhanced recovery protocol (transversus abdominis plane (TAP) blocks with bupivacaine (no epidural analgesia), PCA, enteral feeding on postoperative day-1, removal of urinary catheter on day-1 and early ambulation).

Robotic resections were carried out using the DaVinci Xi system (Intuitive Surgical, Sunnyvale, USA) by surgeons who had achieved their training equivalency certification through the Intuitive Surgical program. The following were standardized: four robotic ports plus one assistant port and a Pfannenstiel incision to establish pneumoperitoneum and extract the specimen. Where appropriate, indocyanine green (ICG) was injected into both ureteric openings via cystoscopy using a Chevassu catheter at the start of the procedure to help identify ureters intra-operatively using ‘Firefly’ (integrated fluorescent imaging technology).

Study design and reporting of outcomes was in line with the STROBE initiative [16].

### Statistical analysis

Statistical analysis was performed using IBM SPSS Statistics (version 29). Tests for normality were performed using Kolmogorov–Smirnov and Shapiro–Wilk tests. Categorical variables were compared by Chi Squared test or Fisher’s exact test (for binary outcomes). Continuous variables were compared by unpaired t-test (parametric data) or Mann-Whitney U test (non-parametric data). Due to prefect separation of the data (all conversions to open surgery occurred in the laparoscopic group), Firth logistic regression (penalized likelihood method) was used to predict conversion rate, with surgical approach, previous abdominal surgery, disease phenotype, BMI and ASA-grade as independent variables (R logistf package) [17]. Ordinal regression was used to predict category of blood loss (< 100 ml, 100–500 ml, > 500 ml) with surgical approach, conversion, previous surgery, BMI and disease phenotype as independent variables. Linear regression was used to predict PCA duration (with surgical approach, conversion and operative time as independent variables) and operative time (with surgical approach, BMI, previous surgery and conversion as independent variables). Complete case analysis with listwise deletion was used for missing data. All data are presented as median (range) or mean (+/−SD) unless stated. Two-tailed tests were used where applicable, with an alpha significance level of 0.05. Values are to two decimal places where appropriate.

## Results

**Fig. 1:**
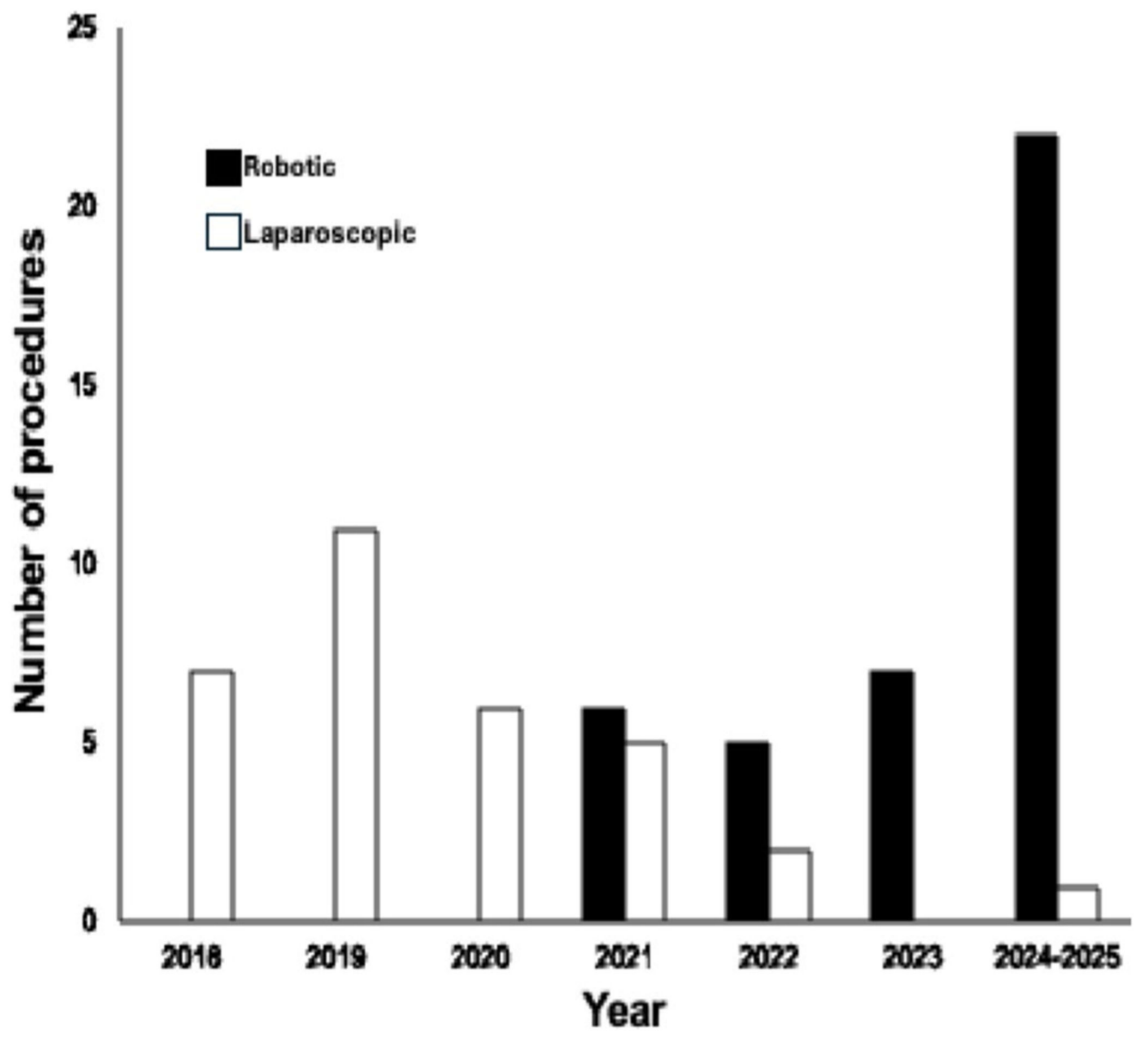
Number of elective robotic and laparoscopic diverticular resections undertaken annually at the study center (2018–2025). The robotic surgery program was initiated in November 2020 with the first diverticular resection in May 2021. From December 2023 all consultant colorectal surgeons had an independent robotic practice

From 1 st February 2018 to 31 st March 2025, 72 patients underwent elective minimally invasive resection of left-sided diverticular disease. The first robotic diverticular resection was completed in May 2021. Figure 1 shows the transition from laparoscopic to robotic surgery during the study period. Of note, all six colorectal surgeons who contributed to the study had an independent robotic practice by December 2023.

Baseline characteristics are documented in Table 1. Robotic and laparoscopic groups were similar in terms of demographic parameters (age, gender, BMI, smoking status) and major comorbidities (hypertension, IHD, COPD, DM). Rates of previous abdominal surgery were 63 and 56%, respectively. A greater proportion of patients had stricturing disease in the robotic group (28 vs. 16%; χ^2^ = 1.45; *p* = 0.23) but fewer had fistulation (33 vs. 56%; χ^2^ = 4.09; *p* = 0.04). Two patients in each group had a defunctioning ileostomy fashioned at a previous operation and attended with this in situ.

85% of patients in the robotic group and 84% in the laparoscopic group had anterior resection, with the remainder having a Hartmann’s procedure. Mean total operative time was 56 min greater in the robotic group (314 ± 79 vs. 258 ± 64 min; *p* < 0.001). Median docking time in the robotic group was 5 min (IQR 3–8 min). Conversion to open surgery was significantly less in the robotic group (0/40 vs. 11/32; χ^2^ = 16.23; *p* < 0.001). Blood loss in the robotic group was significantly less than in the laparoscopic group: 73% and 44% of patients had < 100 ml blood loss; 20% and 31% had 100–500 ml blood loss and; 8% and 6% had > 500 ml blood loss, respectively (χ^2^ = 11.56; *p* = 0.009). For six patients in the laparoscopic group blood loss was not recorded and these cases were excluded from statistical analysis. Of those patients without a pre-existing defunctioning ileostomy, 57 had an anterior resection. In the robotic group, 28/32 were not defunctioned and in the laparoscopic group this proportion was 17/25, which was not statistically different. Duration of PCA was shorter in the robotic group (44 (0–155) vs. 64 (0–358) hours; *p* = 0.03). Median LOS was 5 (2–44) days in the robotic group and 6 (3–38) days in the laparoscopic group, which was not statistically different. Time to gut function was not different between groups. The proportion of graded complications were not significantly different between groups. Serious complications (CD 3–4) occurred in 1/40 robotic and 4/32 laparoscopic cases, which did not reach significance (χ^2^ = 2.75; *p* = 0.10). Need for post-operative critical care, anastomotic leak, return to theatre and 30-day re-admission were not different between groups. 30-day mortality was zero in both groups. Table 2 shows the frequencies of clinical outcomes.

Firth logistic regression showed that the robotic approach was an independent predictor of conversion (Table 3). In this model, the odds of conversion were 3.5 times lower in the robotic group ([−8.15 to −1.45]; *p* < 0.001). The other variables (disease phenotype, previous abdominal surgery, BMI and ASA-grade did not predict for conversion and were therefore not confounders in this model. Ordinal logistic regression showed that the odds of blood loss > 100 ml were 1.4 times lower in the robotic group ([−2.64 to −0.08]; *p* = 0.04) and this was independent of conversion to open, previous abdominal surgery, BMI and disease phenotype, which did not predict blood loss (Table 4). In a linear regression model, operative approach, conversion to open or operative time did not independently predict PCA duration (Table 5). Finally, surgical approach was an independent predictor of higher operative time, with BMI, previous surgery and open conversion not predicting operative time (Table 6). In this linear regression model robotic surgery increased operative time by 44 min compared to laparoscopic surgery.

## Discussion

In the present study, which is the first reported cohort of its kind from the UK, we showed that open conversion, blood loss and PCA use were reduced in patients having robotic compared to laparoscopic left-sided diverticular resection, at the cost of total operative time. Furthermore, robotic surgery was an independent predictor of lower conversion rate, reduced blood loss and higher operative time.

These findings are in keeping with systematic reviews which have shown lower conversion rates but greater operative time in robotic compared to laparoscopic diverticular resections [6–8]. However, as alluded to, the studies included in these meta-analyses are quite heterogenous in terms of disease phenotype. In the Larkins et al. meta-analysis of 3711 robotic cases, one study contributing 1301 robotic cases only had 4% of patients with complicated diverticular disease, and another study (1257 patients), did not document the diverticular phenotype at all [6, 18, 19]. Similarly, in another recent meta-analysis of eight comparative studies in which 1892 patients had robotic diverticular resection, the Raskin et al. cohort, which did not specify disease phenotype, contributed significant weight with over 1200 robotic cases [8, 19]. Giuliani et al. reported on 1922 robotic cases in their meta-analysis of nine comparative studies but one of these studies included patients having emergency surgery and some included patients with uncomplicated disease [7, 20, 21]. In this regard, the disease phenotype of patients in the present study was well documented and all patients who had elective diverticular resection met ESCP criteria: symptomatic chronic inflammation, stricture, ongoing abscess or fistula [9].

Whereas most studies have shown a consistent reduction in conversion rate with robotic compared to laparoscopic diverticular resection, there are fewer studies showing significant results for blood loss. Maciel et al. reported mean estimated blood loss of 100 ml in their robotic group and 190 ml in the laparoscopic group, but their study only focused on patients with colovesical fistulae [22]. More recently, Presl and colleagues showed that postoperative drop in hemoglobin was 1.3 g/dL in their robotic group and 1.8 g/dL in the laparoscopic group, including patients with both acute and chronic diverticulitis [23]. However, when diverticular studies were meta-analyzed, there was no significant difference in blood loss between robotic and laparoscopic groups [8]. Furthermore, in large scale randomized studies such as the REAL trial for rectal cancer, small but statistically significant differences were recorded, with median blood loss 40 ml in the robotic arm and 50 ml in the laparoscopic arm, the clinical significance of which is likely negligeable [24]. Perhaps then, the benefits of robotic surgery in reducing blood loss are relevant only in subgroups of patients, such as those with complex inflammatory pathology [22]. However, intraoperative blood loss is notoriously difficult to quantify accurately and is unreported in a significant number of cases, which may mask the true effect [25].

In the present study there were no difference in LOS, stoma formation and complications between groups. In terms of LOS, the Giuliani meta-analysis shows a small but significant reduction in LOS for patients having robotic surgery, whereas the Eltyeb meta-analysis shows no difference [7, 8]. In the former, a study by Al-Temimi et al. contributes 62.5% weight and shows a mean reduction in LOS of 0.9 days, but in the latter, this study contributes only 20% weight [26]. In both the aforementioned meta-analyses, studies generally show a small reduction in LOS, except for the study by Elliott et al. (fistulating diverticular disease) which showed that mean LOS was more than two days greater in the robotic group [27]. However, it should be noted that in this study, more patients in the robotic group had rectal resections, which may account for this [27]. Eltyeb et al. report on stoma formation, demonstrating no difference between groups [8]. This is understood to be the proportion of patients having any stoma, which in this context is typically a defunctioning loop ileostomy in an anterior resection or an end colostomy in a Hartmann’s procedure. However, we are of the opinion that these should be separate outcomes because the decision to perform a Hartmann’s procedure is often taken pre-operatively, whereas the decision to defunction a left-sided anastomosis is often taken intraoperatively. Therefore, the surgical approach (robotic vs. laparoscopic) will have more of an impact on defunctioning ileostomy rate compared to end colostomy rate. Complication rate in the three systematic reviews, including anastomotic leak rate, was no different between robotic and laparoscopic groups and 30-day mortality was less than 1% [6–8].

In terms of operative time, all published systematic reviews have shown that robotic procedures for diverticular disease take longer than laparoscopic procedures [6–8]. Our study suggests that this is independent of BMI, previous surgery or conversion to open. Liu et al. compared operative time for robotic and laparoscopic gastrectomy and suggested that the difference was due to ‘junk’ time, which is lost during set up of the robot, docking and instrument changes [28]. In our study, we measured total operative time from the first incision to the end of the skin closure. This typically excluded time for robotic set up which occurred during induction of anesthesia. Median docking time in our study was only 5 min and on average, total operative time was 56 min longer for robotic cases. It could be inferred therefore that the added time in robotic procedures was mainly due to instrument changes. However, the more likely explanation is that surgeons were on their robotic learning curve for a significant proportion of cases. We should therefore expect differences in operative time to get smaller with greater surgeon experience. Nonetheless, we may have to accept that robotic surgery takes longer than laparoscopic surgery even after factoring in the learning curve.

Clearly there are concerns about the increased upfront cost of robotic surgery compared to laparoscopic surgery, not only because of increased operative time but also due to the cost of platforms, consumables and maintenance. Khorgami et al. used the US National Inpatient Sample to show that robotic sigmoid colectomy costs $16,652 compared to $13,504 for laparoscopic sigmoid colectomy [29]. Rashidi and colleagues showed that although supply costs for robotic abdominal surgery were higher, total direct costs (theatre supplies, operative time and pre- and postoperative care) were similar to laparoscopic surgery [30]. In any case, any difference in direct costs needs to be weighed up against the reduced conversion rate, which is likely to reduce future adhesional complications and incisional hernia rates (indirect costs).

There are no RCTs on robotic vs. laparoscopic diverticular resection to date and this reflects the difficulty in performing randomized trials for surgical procedures. One of the criticisms of the ROLAAR trial comparing robotic and laparoscopic rectal cancer surgery was that surgeons performing robotic procedures were early in their learning curve whilst those performing laparoscopic procedures were relatively more experienced in that approach [31]. This will apply to most studies on robotic surgery, including the present study, regardless of design, mainly because laparoscopic surgery predates robotic surgery, however, there are novel methods to estimate and adjust for learning effects [32]. Another concern about large scale multicenter trials is that surgical technique and quality are more variable because of the large number of participating surgeons. It is worth remembering that randomization helps equally apportion patient characteristics into different groups, but it does not equally distribute surgeons or variations in procedural technique between groups. In this regard, single center studies may have the advantage that surgical technique is likely to be more standardized, and quality is likely to be less variable. However, the drawback is that findings of smaller single center studies may be less reproducible and generalizable. Although RCTs are methodologically superior in terms of lower systematic error and higher internal validity, they can be challenging to conduct when the intervention is a surgical procedure. Comparative studies remain relevant, particularly in the exploration phase of the IDEAL frame-work and should inform future RCTs [14, 15]. Interestingly, and perhaps due to the challenges outlined above, there are no published or currently recruiting RCTs on robotic diverticular surgery, but the ROLADI study (NCT05829343) is a multicenter prospective cohort study aiming to recruit 1450 patients, which has not yet published outcomes.

The present study has some key advantages, which include prospectively documented outcomes, accurate recording of diverticular disease phenotype and standardized operative technique amongst participating surgeons, however, there are clearly some limitations. The overall number of patients is modest, and this meant that events for certain outcomes (critical care admission, anastomotic leak, RTT and 30-day re-admission) were too small to compare between groups. Case numbers reflect the population that the institution serves as a district general hospital. The unit adheres to current guidelines for elective diverticular resection, which have moved away from operating on patients with uncomplicated disease, thereby narrowing the eligible patient cohort [9, 11]. Furthermore, the study period traverses the Covid-19 pandemic during which the number of elective procedures for diverticular disease were reduced globally [33]. In the post-pandemic era, the focus has been on prioritizing cancer surgery, with subsequent reduction in capacity for benign conditions [34].

The study period spans seven years (2018–2025), which may raise concerns about changes in clinical practice and advances in surgical technology in this time frame. However, there were no significant changes in the recommended indication for elective diverticular resection between the UK ACPGBI guidelines in 2011 and the ESCP guidelines in 2020 [9, 11]. Moreover, the DaVinci Xi platform used in this study, launched in 2014, remained the most widely used across the globe during the study period, without significant advances in robotic technology. For example, Vessel Sealer™ technology (FDA approval 2012) and Sureform™ stapler (FDA approval 2018) both predate the first robotic case in this study. With regard to laparoscopic surgery, it is fair to say that by 2018, when the study commenced, advances in tools and techniques had plateaued, and there were no notable changes in study period.

We should next consider the effect of channeling and performance bias. Channeling bias may have resulted from surgeon selection for robotic or laparoscopic surgery, depending on patient factors, disease phenotype and operative experience but this could be in either direction. For example, a surgeon may prefer to use the robotic platform over laparoscopic surgery for patients with high BMI but the opposite may be true for more complex disease, particularly early in their learning curve [35]. Regarding performance bias, it is possible that surgeons using a novel robotic platform may be less inclined to convert to open surgery as this may be perceived to be a failure. On the other hand, one can argue that early in the robotic learning curve, there may be greater likelihood of converting to a more familiar open approach. Furthermore, only six surgeons contributed cases and this may have magnified these biases.

In the present study, although there was significantly less blood loss in the robotic group, blood loss was unrecorded for six patients in the laparoscopic group. These patients were excluded from statistical analysis. However, if one takes the non-recording of blood loss to mean minimal blood loss, then there would be no significant difference between groups. It is therefore important not to overinterpret the effect of robotic surgery on this parameter.

Clearly, this study focuses on immediate and short-term outcomes. Longer-term outcomes such as bowel function and recurrence of diverticulitis are also important, as are patient-reported outcome measures and quality of life estimates, which could be incorporated into interval analyses of future studies [36, 37].

Overall, in this cohort, robotic diverticular resection offered reduced conversion rate in comparison to laparoscopic resection, at the cost of a greater operative time, and without any difference in complication rate. Therefore, the robotic approach for diverticular disease may enable a greater proportion of patients to benefit from minimally invasive surgery without any detrimental effects.

## Figures and Tables

**Table 1:** Baseline characteristics of the cohort

		Robotic *(n*=40)	Laparoscopic (*n* = 32)	Total (*n* = 72)
Age (years)		60 ± 11	61 ± 11	61 ± 11
Gender (f)		22/40 (55%)	17/32 (53%)	39/72 (54%)
BMI (kg/m^2^)		28.0 ± 5.1	27.7 ± 5.3	27.8 ± 5.2
Current smoker		11/40 (28%)	6/32 (19%)	17/72 (24%)
Hypertension		16/40 (40%)	13/32 (41%)	29/72 (40%)
IHD		2/40 (5%)	2/32 (6%)	4/72 (6%)
COPD		3/40 (8%)	3/32 (9%)	6/72 (8%)
DM		1/40 (3%)	4/32 (13%)	5/72 (7%)
ASA grade	1	2/40 (5%)	0/32	2/72 (3%)
	2	29/40 (73%)	26/32 (81%)	55/72 (76%)
	3	9/40 (23%)	6/32 (19%)	15/72 (21%)
	4	0/40	0/32	0/72
Previous abdominal surgery		25/40 (63%)	18/32 (56%)	43/72 (60%)
Diverticular phenotype	Chronic inflammation	13/40 (33%)	7/32 (22%)	20/72 (28%)
	Stricture	11/40 (28%)	5/32 (16%)	16/72 (22%)
	Abscess	3/40 (8%)	2/32 (6%)	5/72 (7%)
	Fistula	13/40 (33%)	18/32 (56%)	31/72 (43%)
Existing defunctioning ileostomy		2/40 (5%)	2/32 (6%)	4/72 (6%)

*BMI* body mass index; *IHD* ischemic heart disease (objective evidence of coronary artery disease); *COPD* chronic obstructive pulmonary disease; *DM* diabetes mellitus;ASA American Society of Anesthesiologists. Values are presented as mean +/−SD, median (range) or frequencies

**Table 2:** A comparison of primary and secondary outcomes

		Robotic	Laparoscopic	Test statistic	*P*
Operative time (min)		314 ± 79	258 ± 64	t = –3.33	*0.001*
Docking time (min)		5 (3–8)	*n/a*	*n/a*	*n/a*
Open conversion		0/40	11/32 (34%)	χ^2^ = 16.23	*0.001*
Blood loss	< 100 ml	29/40 (73%)	14/32 (44%)	χ^2^ = 11.56	*0.009*
	100–500 ml	8/40 (20%)	10/32 (31%)		
	> 500 ml	3/40 (8%)	2/32 (6%)		
	Unrecorded	0/40	6/32 (19%)		
Stoma formation	Defunctioning ileostomy*	4/32	8/25	χ^2^ = 3.21	*0.07*
	End colostomy	6/40	5/32	χ^2^ = 0.0054	*0.94*
PCA duration (h)		44(0–155)	64 (0–358)	*U* = 358.00	*0.03*
Time to gut function (days)		3 (1–8)	3(1–12)	*U* = 538.00	*0.68*
LOS (days)		5 (2–44)	6 (3–38)	*U* = 549.50	*0.30*
Critical care		1/40 (3%)	3/32 (9%)	χ^2^ = 1.60	*0.21*
Complications	CD 0	33/40 (80%)	20/32 (63%)	χ^2^ = 5.58	*0.23*
	CD 1	4/40 (10%)	5/32 (16%)		
	CD 2	2/40 (5%)	2/32 (6%)		
	CD 3	0/40	3/32 (9%)		
	CD 4	1/40 (3%)	1/32 (6%)		
Anastomotic leak^†^		1/34 (3%)	2/27 (7%)	χ^2^ =0.64	*0.42*
Return to theatre		2/40 (5%)	2/32 (6%)	χ^2^ = 0.05	*0.82*
30-day re-admission		2/40 (5%)	1/32 (3%)	χ^2^ = 0.194	*0.66*

* Patients who had a pre-existing defunctioning ileostomy or had a Hartmann’s procedure were excluded from this analysis

† Patients who had a Hartmann’s procedure were excluded from this analysis. CD Clavien Dindo

**Table 3:** Firth regression for predicting conversion to open surgery

Variable	Reference	Predictor	Coefficient	S.E.	95% CI Lower	95% CI Upper	χ^2^	*P*
Surgical approach	Laparoscopic	Robotic	–3.49	1.17	–8.15	–1.45	14.99	*< 0.001*
Disease phenotype	Chronic inflammation	Stricture	–0.13	1.15	–2.82	2.39	0.01	*0.92*
		Abscess	1.98	1.52	–1.34	7.16	1.30	*0.26*
		Fistula	–0.60	0.83	–2.32	1.16	0.48	*0.49*
Previous surgery	No	Yes	–0.28	0.73	–1.81	1.30	0.13	*0.72*
BMI (kg/m^2^)	N/A	BMI	–0.07	0.07	–0.23	0.08	0.75	*0.39*
ASA-grade	ASA1	ASA-2	–2.38	2.04	–7.75	3.04	1.04	*0.31*
		ASA-3	–2.28	2.08	–7.64	3.20	0.93	*0.34*

The dependent variable was open conversion. The estimation method was Firth penalized maximum likelihood. Likelihood ratio test = −19.96, degrees of freedom 8. SE standard error. BMI body mass index. CI confidence interval. P statistical significance

**Table 4:** Ordinal regression for predicting intraoperative blood loss

Variable	Reference	Predictor	Estimate	S.E.	Wald	df	*P*	95% CI Upper	95% CI Lower
Surgical approach	Laparoscopic	Robotic	–1.36	0.65	4.31	1	*0.04*	–2.64	–0.08
Conversion	No	Yes	0.74	0.77	0.93	1	*0.33*	–0.76	2.25
Disease phenotype	Chronic inflammation	Stricture	0.94	0.67	1.97	1	*0.16*	–0.37	2.25
		Abscess	0.53	1.04	0.27	1	*0.61*	–1.50	2.56
		Fistula	–0.65	0.90	0.51	1	*0.48*	–2.42	1.12
Previous surgery	No	Yes	0.14	0.55	0.06	1	*0.81*	–0.95	1.22
BMI (kg/m^2^)	N/A	BMI	0.07	0.06	1.84	1	*0.18*	–0.03	0.18
Operative time (min)	N/A	Operative Time	0.01	0.004	1.86	1	*0.17*	–0.002	0.01

The dependent variable was blood loss (<100, 100-500 or >500 ml). The predictors were: surgical approach, open conversion, previous abdominal surgery, BMI and disease phenotype. SE standard error. df degrees of freedom. P statistical significance. BMI body mass index. CI confidence interval

**Table 5:** Linear regression model for predicting postoperative PCA duration

Variable	B	S.E.	Beta	t	*P*	95% CI Upper	95% CI Lower
Surgical approach	–22.04	17.02	–0.16	–1.30	0.20	–56.08	12.00
Conversion	29.81	22.94	0.18	1.30	0.20	–16.08	75.69
Operative time (min)	0.01	0.10	0.01	0.06	0.96	–0.20	0.21

The dependent variable was PCA duration (h). B is the unstandardized coefficient, and beta is the standardized coefficient. SE standard error. t Test statistic. P statistical significance. CI confidence interval

**Table 6:** Linear regression model for predicting operative time

Variable	B	S.E.	Beta	t	*P*	95% CI Upper	95% CI Lower
Surgical approach	43.84	20.19	0.28	2.17	0.03	3.53	84.16
BMI (kg/m^2^)	0.73	1.75	0.05	0.42	0.68	–2.77	4.23
Previous surgery	12.52	18.39	0.08	0.68	0.49	–24.20	49.25
Conversion	–31.78	27.97	–0.15	–1.14	0.26	–87.64	24.09

The dependent variable was operative time (min). B is the unstandardized coefficient, and beta is the standardized coefficient. SE standard error. t Test statistic. P statistical significance. BMI body mass index. CI confidence interval

## Data Availability

All data supporting the findings of this study are available within the paper and its Supplementary Information.
